# Troponin Levels and the Severity of COVID-19 Pneumonia

**DOI:** 10.7759/cureus.23193

**Published:** 2022-03-15

**Authors:** Turki Alhindi, Hamza Awad, Dunya Alfaraj, Salih Elabdein Salih, Maged Abdelmoaty, Aroub Muammar

**Affiliations:** 1 Emergency Department, King Fahad Specialist Hospital, Dammam, SAU; 2 Emergency Department, King Fahad General Hospital, Al Madinah, SAU; 3 Emergency Department, King Fahad University Hospital, Imam Abdulrahman Bin Faisal University, Dammam, SAU; 4 Emergency Department, King Fahad University Hospital, Al Khobar, SAU; 5 Emergency Department, Imam Abdulrahman Bin Faisal University, Dammam, SAU

**Keywords:** prognostic, severity, pneumonia, covid-19, troponin

## Abstract

Introduction

In late 2019, a novel coronavirus was identified as the pathogen responsible for a cluster of pneumonia cases in Wuhan, a city in the Hubei Province of China. Elevated cardiac troponin is a marker of myocardial injury, which is commonly seen in hospitalized patients with COVID-19 due to unclear reasons. The frequency of elevated troponin levels in patients with COVID-19 is variable and is reported in up to 7-36% of patients. The troponin level may be associated with the severity of COVID-19, and mild cases of COVID-19 tend to have a normal troponin level. This study aims to determine the frequency of patients with COVID-19 who had elevated troponin levels on presentation to the ED and determine the factors associated with elevated troponin levels. Additionally, the study aims to identify the association of elevated troponin and the outcome of COVID-19.

Methodology

A retrospective study wherein the factors associated with elevated troponin levels in COVID-19 pneumonia were evaluated. The study was conducted in King Fahd Hospital of the Imam Abdulrahman Bin Faisal University. The Hospital Information System was used to identify all visits to the ED from March 2020 to November 2020 for patients who tested positive for SARS-CoV-2. In addition, a structured data collection form was used to collect data from the electronic health records. The data collection was conducted by emergency medicine physicians who were given a detailed explanation of the purpose of the study and had training and supervision by the principal investigator.

Results

The study involved 214 patients who presented to the ED and had positive results on the SARS-CoV-2 reverse transcription polymerase chain reaction (RT-PCR) test and had troponin-I levels measured. Patients with elevated troponin levels were more likely to require supplementary oxygen compared with those with normal troponin levels (88.0 vs. 58.5%; *P* < 0.01). In total, 36 (76.6%) patients with elevated troponin levels required admission to the ICU compared with 58 (45.0%) patients with normal troponin levels (*P *< 0.01). Multivariable binary logistic regression analysis was used to identify the predictors of elevated troponin levels on presentation. The model revealed that being admitted in the ICU was the single independent predictor (*P* = 0.02).

Conclusion

The study demonstrated that the troponin level on presentation to the ED was a viable independent prognostic factor in COVID-19 pneumonia. However, further studies are needed to investigate targeted therapeutic interventions among patients with elevated troponin levels, such as cardioprotective therapies like corticosteroids, immunosuppressants, antivirals, or immunoglobulins.

## Introduction

In late 2019, a novel coronavirus was identified as the pathogen responsible for a cluster of pneumonia cases in Wuhan, a city in the Hubei Province of China. This coronavirus disease 2019 (COVID-19) has spread rapidly in China and other countries, resulting in a pandemic declared by the WHO on March 11, 2020 [1].

The spectrum of clinical severity of COVID-19 is very broad. While the clinical manifestations of COVID-19 have been characterized by cough, fever, myalgia, and headache, extrapulmonary involvement has also been frequently observed [2]. Cardiac involvement in COVID-19 has a wide spectrum. For example, some patients have no clinical evidence of cardiac disease, some have no cardiac symptoms but have cardiac test abnormalities (such as elevated cardiac enzymes or abnormal imaging findings), and some have symptomatic cardiac disease, including myocardial injury, cardiac failure, and arrhythmias [3].

Elevated cardiac troponin is a marker of myocardial injury, commonly seen in hospitalized patients with COVID-19 due to unclear reasons [3]. The frequency of elevated troponin levels in patients with COVID-19 is variable and is reported in up to 7-36% of patients [4]. However, since the studies have used different troponin assays, upper reference limit, and sampling times, it is difficult to allow for comparison among such studies [5]. In addition, the troponin level may be associated with the severity of COVID-19, and mild cases of COVID-19 tend to have a normal troponin level [6].

This study aims to determine the frequency of patients with COVID-19 who had elevated troponin levels on presentation to the ED and determine the factors associated with elevated troponin levels. Additionally, the study aims to identify the association of elevated troponin and the outcome of COVID-19.

## Materials and methods

Study design

After obtaining an ethical review board approval, we conducted a retrospective study wherein the factors associated with elevated troponin levels in COVID-19 pneumonia were evaluated.

Study setting

The study was conducted in King Fahad Hospital of the Imam Abdulrahman Bin Faisal University, the single academic hospital in the Eastern Province of Saudi Arabia. It has around 250,000 visits to the ED annually.

Study population

The Hospital Information System was used to identify all visits to the ED from March 2020 to November 2020 for patients who tested positive for SARS-CoV-2 and underwent frontal chest X-ray on their presentation. Patients below the age of 18 years and pregnant patients were excluded.

Study objectives

The study aimed to identify the demographic, clinical, and laboratory parameters associated with elevated troponin levels in COVID-19 pneumonia on presentation. Additionally, it investigated the association of elevated troponin and the outcome of COVID-19, including the need for intensive care, mechanical ventilation, and in-hospital mortality.

Data collection

A structured data collection form was used to collect data from the electronic health records. The data collection was conducted by emergency medicine physicians who were given a detailed explanation of the purpose of the study and had training and supervision by the principal investigator. The collected data included age, sex, comorbidities, presenting symptoms, duration of symptoms, and vital signs. In addition, data on the severity of COVID-19 pneumonia were collected, including the disposition decision (discharge, admission, or referral), the need for mechanical ventilation, the need for ICU admission, thrombotic complication, length of stay, and mortality. The Charlson Comorbidity Index was used to measure comorbidities. This index score ranges from 0 to 37 and includes various medical conditions [7].

Ethical consideration

The study was approved by the Institutional Review Board at the Imam Abdulrahman bin Faisal University (IAU IRB No. 2020-01-384). The data were stored electronically, and only the principal investigator had access to the stored data. Furthermore, the need for informed consent was waived because the patients were de-identified.

Statistical analysis

After checking for completeness and consistency, data were analyzed using IBM SPSS for Windows, version 26 (IBM Corp., Armonk, NY, USA). Categorical variables, presented as percentages and frequency distribution, were compared using the Chi-squared or Fisher’s exact tests. Continuous variables, presented as mean and SD, were compared using the independent sample t-test or ANOVA. Multivariable binary logistic regression analysis was conducted to identify the independent factors associated with elevated troponin. Candidate variables were selected based on medical literature and bivariate analyses. OR with 95% CI were estimated using the full model fit and were reported in comparison with the designated reference group. The presence of multicollinearity was assessed using the bivariate Spearman’s correlation coefficients. The goodness-of-fit of the model was evaluated using the Omnibus and Hosmer-Lemeshow tests. The significance level was defined as α = 0.05.

## Results

Patient characteristics

The study involved 214 patients who presented to the ED and had positive results on the SARS-CoV-2 reverse transcription polymerase chain reaction (RT-PCR) test and had troponin-I levels measured. This included 148 (69.2%) men and 66 (30.8%) women. Overall, 92 (43.0%) were aged 51-65 years, and 50 (23.4%) were aged above 65 years. In total, 89 (41.6%) patients had a Charlson Comorbidity Index score of 0-1, whereas 38 (17.8%) patients had a score of 4 or more. Diabetes mellitus and hypertension were the most frequent comorbidities, with 96 (44.4%) and 95 (44.4%) patients, respectively. In addition, asthma or chronic obstructive pulmonary disease were seen in 22 (10.3%) patients. Dyspnea (65.9%), fever (54.7%), and cough (51.4%) were the most frequently reported symptoms. Nearly half (48.6%) of patients had an oxygen saturation below 95%, and 147 (68.7%) had a respiratory rate above 20 bpm (Table 1).

**Table 1:** Characteristics of patients. N: Number of patients; COPD: Chronic obstructive pulmonary disease; URT: Upper respiratory tract.

Variables		N	(%)
Age	18-35 years	21	(9.8)
	36-50 years	51	(23.8)
	51-65 years	92	(43.0)
	>65 years	50	(23.4)
Gender	Male	148	(69.2)
	Female	66	(30.8)
Charlson Comorbidity Index	0-1	89	(41.6)
	2-3	87	(40.7)
	≥ 4	38	(17.8)
Comorbidities	Hypertension	95	(44.4)
	Diabetes mellitus	96	(44.9)
	Ischemic heart disease	20	(9.3)
	Asthma/COPD	22	(10.3)
	Chronic kidney disease	11	(5.1)
	Malignancy	2	(0.9)
Presenting symptoms	Fever	117	(54.7)
	Dyspnea	141	(65.9)
	Cough	110	(51.4)
	Nausea/Vomiting	24	(11.2)
	Fatigue	21	(9.8)
	Diarrhea	15	(7.0)
	Abdominal pain	18	(8.4)
	Headache	12	(5.6)
	Myalgia	5	(2.3)
	URT symptoms	11	(5.1)
	Loss of taste or smell	6	(2.8)
Duration of symptoms	1 day	34	(17.1)
	2-3 days	59	(29.6)
	4-7 days	64	(32.2)
	>7 days	42	(21.1)
Vital signs	Heart rate >100 bpm	92	(43.0)
	Respiratory rate >20 bpm	147	(68.7)
	Temperature ≥38°	57	(26.6)
	Oxygen saturation <95%	104	(48.6)

Hospital course of the disease

Table 2 summarizes the hospital course of the patients in the study. Most (82.2%) patients were admitted. The median length of stay of such patients was 11.0 days. Nearly one-third (34.6%) of patients did not need supplementary oxygen during their hospital stay. However, 50 (23.4%) patients required intubation and mechanical ventilation. Over half (53.4%) of patients required admission to the ICU. Six patients developed thrombotic complications, including myocardial infarction (n=3), stroke (n=2), and pulmonary embolism (n=2). In total, 44 (20.6%) patients died during hospitalization.

**Table 2:** Hospital course of the patients. N: Number of patients; IQR: Interquartile range.

Variables		N	(%)
Supplementary oxygen	Not needed	74	(34.6)
	Mechanical ventilation	50	(23.4)
Disposition decision	Discharged	32	(15.0)
	Transferred	6	(2.8)
	Admitted	176	(82.2)
Need for intensive care	Yes	94	(53.4)
	No	82	(38.3)
Thrombotic complications	Yes	6	(2.8)
	No	208	(97.2)
Outcome	Recovered	170	(79.4)
	Died	44	(20.6)
Length of stay (days)	Median (IQR)	11.0 (6.0-20.5)	

Factors associated with elevated troponin levels

Overall, 50 (23.4%) patients had elevated troponin levels on presentation. The age and gender of patients were not significantly associated with elevated troponin levels (P > 0.05). However, the Charlson Comorbidity Index score was significantly associated with troponin levels (P = 0.02). For instance, 74 (83.1%) patients with a score of 0-1 had normal troponin levels, whereas 15 (39.5%) patients with a score of 4 and above had elevated troponin levels. Among the comorbidities, the history of chronic kidney disease was the only comorbidity associated with elevated troponin levels (P < 0.01). More patients with chronic kidney disease had elevated troponin levels than those who had not (63.6% vs. 21.2%). There was no significant association between the duration of symptoms and the troponin levels (P = 0.12) (Table 3).

**Table 3:** Factors associated with elevated troponin levels on presentation. N: Number of patients; COPD: Chronic obstructive pulmonary disease; URT: Upper respiratory tract.

Variables		Normal troponin		Elevated troponin		P-value
		N	(%)	N	(%)	
Age	18-35 years	17	(81.0)	4	(19.0)	0.245
	36-50 years	41	(80.4)	10	(19.6)	
	51-65 years	73	(79.3)	19	(20.7)	
	>65 years	33	(66.0)	17	(34.0)	
Gender	Male	110	(74.3)	38	(25.7)	0.232
	Female	54	(81.8)	12	(18.2)	
Charlson Comorbidity Index	0-1	74	(83.1)	15	(16.9)	0.022
	2-3	67	(77.0)	20	(23.0)	
	≥4	23	(60.5)	15	(39.5)	
Comorbidities	Hypertension	68	(71.6)	27	(28.4)	0.118
	Diabetes mellitus	69	(71.9)	27	(28.1)	0.138
	Ischemic heart disease	15	(75.0)	5	(25.0)	0.856
	Asthma/COPD	19	(86.4)	3	(13.6)	0.255
	Chronic kidney disease	4	(36.4)	7	(63.6)	0.004
	Malignancy	2	(100.0)	0	(0.0)	1.000
Presenting symptoms	Fever	92	(78.6)	25	(21.4)	0.448
	Dyspnea	107	(75.9)	34	(24.1)	0.719
	Cough	86	(78.2)	24	(21.8)	0.582
	Nausea/Vomiting	21	(87.5)	3	(12.5)	0.182
	Fatigue	18	(85.7)	3	(14.3)	0.301
	Diarrhea	15	(100.0)	0	(0.0)	0.027
	Abdominal pain	17	(94.4)	1	(5.6)	0.062
	Headache	12	(100.0)	0	(0.0)	0.049
	Myalgia	5	(100.0)	0	(0.0)	0.212
	URT symptoms	10	(90.9)	1	(9.1)	0.251
	Loss of taste or smell	5	(83.3)	1	(16.7)	0.694
Duration of symptoms	1 day	23	(67.6)	11	(32.4)	0.115
	2-3 days	49	(83.1)	10	(16.9)	
	4-7 days	52	(81.3)	10	(16.9)	
	> 7 days	28	(66.7)	14	(33.3)	
Vital signs	Heart rate >100 bpm	66	(71.7)	24	(19.3)	0.142
	Respiratory rate >20 bpm	103	(70.1)	44	(29.9)	0.001
	Temperature ≥38°	44	(77.2)	13	(22.8)	0.908
	Oxygen saturation <95%	77	(74.0)	27	(26.0)	0.384

Regarding the hospital course of the disease, patients with elevated troponin levels were more likely to require supplementary oxygen compared with those with normal troponin levels (88.0 vs. 58.5%; P < 0.01). Similarly, 19 (38.0%) patients with elevated troponin levels required mechanical ventilation compared with 31 (18.9%) patients with normal troponin levels (P = 0.01). In total, 36 (76.6%) patients with elevated troponin levels required admission to the ICU compared with 58 (45.0%) patients with normal troponin levels (P < 0.01). In addition, the mortality rate during hospitalization among patients with elevated troponin levels was higher than those with normal troponin levels (36.0% vs. 15.9%; P < 0.01). However, the troponin levels were not found to be significantly associated with the length of stay or the occurrence of thrombotic complications (P > 0.05) (Table 4).

**Table 4:** Hospital course of the patients. N: Number of patients; IQR: Interquartile range.

Variables		Normal troponin		Elevated troponin		P-value
		N	(%)	N	(%)	
Supplementary oxygen	Not needed	68	(91.9)	6	(8.1)	<0.001
	Mechanical ventilation	31	(62.0)	19	(38.0)	0.005
Disposition decision	Discharged	29	(90.6)	3	(9.4)	0.040
	Transferred	6	(100.0)	0	(0.0)	
	Admitted	129	(73.3)	47	(26.7)	
Need for intensive care	Yes	58	(61.7)	36	(38.3)	<0.001
	No	71	(86.6)	11	(13.4)	
Thrombotic complications	Yes	4	(66.7)	2	(33.3)	0.558
	No	160	(76.9)	48	(23.1)	
Outcome	Recovered	138	(81.2)	32	(18.8)	0.002
	Died	26	(59.1)	18	(40.9)	
Length of stay (days)	Median (IQR)	11.0 (6.3-21.0)		9.0 (2.0-17.5)		0.072

Multivariable analysis of factors associated with elevated troponin levels

Multivariable binary logistic regression analysis was used to identify the predictors of elevated troponin levels on presentation. The model revealed that being admitted in the ICU was the single independent predictor (P = 0.02). For example, patients who were admitted to the ICU were 3.3-times more likely to have elevated troponin levels compared with those who did not (OR = 3.3; 95% CI: 1.2-8.7) (Table 5).

**Table 5:** Multivariable analysis of factors associated with elevated troponin levels.

Variable		Univariable model		Multivariable model	
		OR [95% CI]	P-value	OR [95% CI]	P-value
Age (years)	18–35	Reference group			
	36–50	1.0 [0.3-3.8]	0.956	0.3 [0.1-1.6]	0.163
	51–65	1.1 [0.3-3.7]	0.869	0.2 [0.1-1.1]	0.061
	> 65	2.2 [0.6-7.5]	0.214	0.3 [0.1-2.0]	0.190
Gender	Female	Reference group			
	Male	1.6 [0.8-3.2]	0.234	2.0 [0.8-4.8]	0.145
Charlson Comorbidity Index	0–1	Reference group			
	2–3	1.5 [0.7-3.1]	0.310	1.4 [0.4-5.3]	0.577
	≥ 4	3.2 [1.4-7.6]	0.007	1.6 [0.3-9.3]	0.611
Hypertension		1.7 [0.9-3.1]	0.120	1.3 [0.6-2.9]	0.532
Diabetes mellitus		1.6 [0.9-3.1]	0.139	1.0 [0.4-2.4]	0.977
Chronic kidney disease		6.5 [1.8-23.3]	0.004	2.9 [0.5-17.6]	0.258
Supplementary oxygen	Not Needed	0.2 [0.1-0.5]	<0.001	0.5 [0.1-1.7]	0.254
	Mechanical Ventilation	2.6 [1.3-5.3]	0.006	0.8 [0.3-2.0]	0.648
Need for intensive care		4.0 [1.9-8.6]	<0.001	3.3 [1.2-8.7]	0.018
Thrombotic complications		1.7 [0.3-9.4]	0.562	1.1 [0.2-8.7]	0.945
Died		3.0 [1.5-6.1]	0.003	1.3 [0.5-3.3]	0.520

## Discussion

The study aimed to identify the demographic and clinical factors associated with elevated troponin levels and investigate the association of troponin levels and the outcome of COVID-19 pneumonia. The findings demonstrated that elevated troponin levels were significantly and strongly associated with mortality, the need for ICU, and mechanical ventilation.

Our findings were consistent with previous research, which investigated the association of troponin and the outcome in COVID-19 pneumonia. For example, a meta-analysis showed that elevated troponin is strongly associated with death, severe presentation, hospitalization in the ICU, and/or mechanical ventilation. Furthermore, patients with elevated troponin were 10-times more likely to develop these adverse outcomes [8].

In a study involving over 2500 hospitalized patients with COVID-19, it was found that 36% of patients had elevated troponin levels. The elevated troponin levels were more common among patients with previous cardiovascular disease or having cardiac risk factors. Furthermore, it was found that even mildly elevated troponin levels were significantly associated with mortality. Interestingly, greater elevations of troponin levels were associated with a higher risk of mortality [9].

In a retrospective study of 416 patients with COVID-19, it was found that elevated troponin levels were more prevalent among older patients with comorbidities and greater laboratory abnormalities, and extensive radiographic involvement of the disease. Additionally, the risk of death starting from the time of symptom onset was more than four times higher in patients with evidence of myocardial injury on admission [4].

Although the sex of the patient was not significantly associated with elevated troponin levels among patients with COVID-19 in our study, it is worth noting that there is a notable difference in the upper limit of the normal range of troponin among men and women [10]. Hence, it may be essential to use sex-specific cut-off values in stratifying the severity of COVID-19 based on the troponin levels. 

This is among a few studies to investigate the demographic and clinical findings concerning the initial troponin levels of patients on their presentation to the ED in Saudi Arabia. However, the study has certain limitations. While it included a relatively large sample size, the study was conducted in a single academic institution in the Eastern Province. The retrospective nature of the study is an important limitation.

## Conclusions

The study demonstrated that the troponin level on presentation to the ED was a viable independent prognostic factor in COVID-19 pneumonia. However, further studies are needed to investigate targeted therapeutic interventions among patients with elevated troponin levels, such as cardioprotective therapies like corticosteroids, immunosuppressants, antivirals, and/or immunoglobulins.
